# Guidewire Retention by the Venous Cannula of Veno-Arterial Extracorporeal Membrane Oxygenation

**DOI:** 10.3390/reports7020043

**Published:** 2024-06-04

**Authors:** Sebastian Bratke, Jan A. Graw

**Affiliations:** Department of Anesthesiology and Intensive Care Medicine, Ulm University Medical Center, Ulm University, 89081 Ulm, Germany; sebastian.bratke@uniklinik-ulm.de

**Keywords:** guidewire, Seldinger, central venous catheter, extracorporeal membrane oxygenation, ECMO

## Abstract

While insertion of a central venous catheter (CVC) for intravascular access, diagnosis, and intensive care medical treatment is frequently needed in critically ill patients, retention of the guidewire used for CVC placement with Seldinger’s technique is a very rare complication. In patients treated with extracorporeal membrane oxygenation (ECMO), significant negative pressures in the thoracal and abdominal venous system are generated by the venous ECMO drainage cannula. Therefore, during CVC placement in patients treated with ECMO, special vigilance is required because the significant negative pressures generated by the venous ECMO drainage cannula facilitate venous suction of an unsecured guidewire.

**Figure 1:**
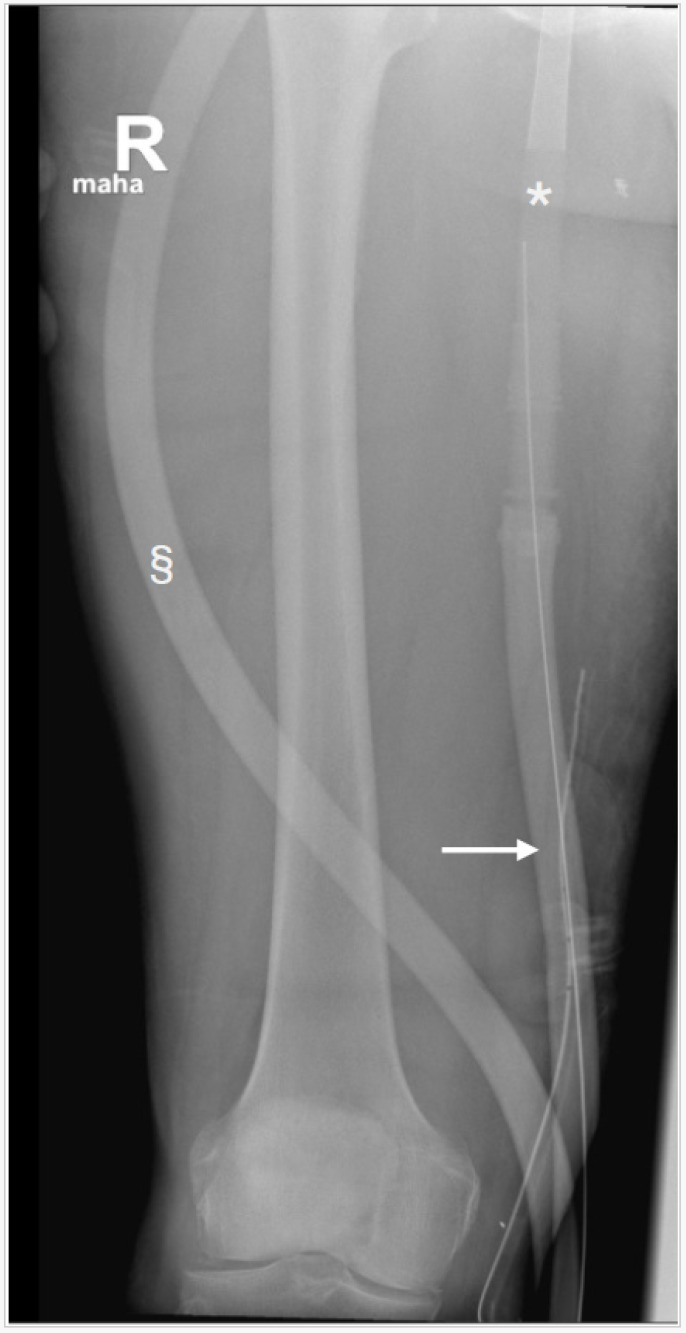
The image was obtained from a patient treated with veno-arterial extracorporeal membrane oxygenation (ECMO) for cardiogenic shock after ST-elevation myocardial infarction and acute coronary artery bypass surgery. Extracorporeal membrane oxygenation (ECMO) is increasingly used in patients with both, respiratory and cardiac failure. For patients with significantly impaired cardiac function, a veno-arterial configuration of ECMO-cannulas is necessary [1]. In the above patient, postoperatively, a second central venous catheter for continuous veno-venous hemofiltration was inserted ultrasound-guided via the right jugular vein using Seldinger’s technique. After puncture of the vein, the guidewire was inserted over the needle with another sonographic position check. During this procedure, the guidewire was aspirated into the venous system. On a consecutive chest X-ray, the lost guidewire was not visible. However, this X-ray of the right thigh indicated aspiration of the guidewire (→) by the venous ECMO cannula (*). § indicates the arterial ECMO circuit tubing. In the aftermath, the guidewire migrated into the venous ECMO tubing until the tip of the guidewire was withheld by the ECMO membrane. ECMO blood flow and function were not affected by the foreign material in the ECMO circuit. The guidewire was easily removed with an exchange of the ECMO system. While insertion of a central venous catheter (CVC) is a frequent procedure in critically ill patients, CVC placement itself is associated with several, mostly iatrogenic, complications [2]. Of those, intravascular retention of the guidewire used for CVC placement with Seldinger’s technique is a very rare but always avoidable complication. Loss of a guidewire in the vasculature must never occur. Because the venous drainage cannula of an ECMO circuit generates significant negative pressures, special vigilance to prevent venous suction of an unsecured guidewire is required in patients treated with ECMO. Adherence to standardized protocols and operating procedures is a measure used to prevent any loss of a guidewire.

## Data Availability

Data are available from the corresponding author on reasonable request.
